# Early evidence for the benefits of biochar in organic regenerative agriculture

**DOI:** 10.1038/s41598-026-40280-5

**Published:** 2026-02-26

**Authors:** L. Kohl, E.-M. L. Minarsch, W. Niether, B. A. Dix, C. Kammann, J. C. Clifton-Brown, A. Gattinger

**Affiliations:** 1 Department of Agronomy and Plant Breeding II, Organic Farming with Focus on Sustainable Soil Use, University of Giessen, Giessen, Germany; 2 Department of Agronomy and Plant Breeding II, Crop Biomass and Bioresources, University of Giessen, Giessen, Germany; 3 Department of Applied Ecology, Hochschule Geisenheim University, Geisenheim, Germany

**Keywords:** Soil organic carbon, Regenerative farming, Biochar, Cover crops, Reduced tillage, Agroecology, Environmental impact

## Abstract

Enhancing soil carbon stocks is important to improve soil quality, but also plays a crucial role in mitigating climate change. The potential of innovative approaches such as regenerative farming practices for increasing soil organic carbon (SOC) needs to be explored. A randomized block experiment was established on an organic farm in Hesse, Germany, to assess the effects of different regenerative agricultural (RA) practices on SOC stock changes over a period of three years (2020–2023). The treatments included minimum tillage combined with cover and nurse crops (RA), RA practices plus the incorporation of biochar (BC) at 30 cm depth with a subsoil loosening device (RABC) and conventional soil cultivation with ploughing and moderate cover cropping as a control. In the beginning and at the end of the experiment, intact soil cores were extracted down to 100 cm depth with a percussion corer and divided into five depth increments for analysis to evaluate changes in SOC stocks. The RABC treatment resulted in the highest increase in native SOC (+ 2.24 Mg C ha^−1^ over three years), in addition to the applied biochar carbon (2.2 Mg C ha^−1^), compared to the control. In contrast, RA alone did not significantly alter SOC stocks compared to the control. Changes in bulk density played a key role in the observed SOC stock differences, with RABC showing the strongest reduction, particularly in deeper layers. Early indicators of SOC stock changes, such as CO_2_-C respiration, water-extractable organic carbon (WEOC), and water-extractable organic nitrogen (WEON), showed positive trends favoring RA and RABC, but effects were not statistically significant. Microbial Biomass Carbon (MBC) in the 0–10 cm soil layer was the strongest early indicator, significantly increasing in both RA and RABC compared to the control. These findings highlight that RA practices, particularly when combined with biochar application in the subsoil, improve soil structure in the early phase after management change and may enhance SOC stocks. However, field experiments lasting more than a decade and full carbon balance assessments are required to evaluate the overall C (CO_2_eq-)sequestration potential and climate mitigation effects including non-CO_2_ greenhouse gas fluxes.

## Introduction

Climate change requires novel management practices in agriculture. Both, climate change mitigation and adaptation, are essential for ensuring food security for future generations^1^. Various arable soil management strategies are discussed for soil organic carbon (SOC) sequestration. These include improved crop rotations and residue return^2^, systematic cover cropping^3^, conservation tillage and the application of organic amendments (e.g.^4^). The addition of (imported) organic carbon does not by itself represent a net sequestration from the atmosphere, especially when the applied amount of carbon exceeds the amount of biomass which could theoretically be produced on-site^5^. Furthermore, the carbon sequestration potential of conservation tillage—referring to reduced or no-till systems that minimize soil disturbance and retain crop residues on the surface—remains controversial, as carbon gains in the topsoil are often offset by losses in the subsoil. For example, in an organic field trial, reduced tillage in organic farming increased SOC stocks in the upper soil layers, but these gains were partially compensated by SOC losses in intermediate depths, resulting in only moderate net increases in total SOC stocks^6^. Organic farming as a system approach includes, to a great extent, the mentioned practices to maximise SOC sequestration. A global meta-analysis from temperate regions found that organic farming increased SOC stocks in the topsoil (0–20 cm) by on average 3.5 Mg C ha^−1^ across 204 paired comparisons. When limiting the analysis to the subset of 41 studies that reported both baseline and final values (thus enabling calculation of annual change), the average SOC sequestration rate was 0.45 Mg C ha^−1^ yr^−1^ compared to non-organic management^5^. However, SOC stocks are potentially at risk of decline in organic farming when cover crops and the retention of residues are not systematically applied^7^.

Cover cropping has been shown to enhance SOC primarily through increased root biomass inputs and stimulation of microbial processes, as root-derived carbon is a key precursor for stable soil organic matter formation^3,8^. A meta-analysis by Poeplau & Don^3^ quantified SOC stock increases under cover cropping at an average rate of + 0.32 Mg C ha^−1^ yr^−1^ across 131 paired comparisons in temperate agricultural soils.

Biochar has also gained attention as a complementary amendment to accelerate SOC accumulation. In a six-year field experiment, Blanco‐Canqui et al.^9^ reported an average SOC stock increase in three no-till cropping systems (Maize; Switchgrass; low-input bioenergy crops) of + 14.1 Mg C ha^−1^ following a single application of 9.3 Mg ha^−1^ biochar (containing 7.25 Mg C ha^−1^), equivalent to nearly double the applied amount of biochar-C, compared with an increase of only 2.3 Mg C ha^−1^ in the control treatment; the authors attribute this to a negative priming effect on the mineralization of native soil organic carbon. Also, Weng et al.^10^ observed a significant, repeated increase of the soil organic carbon content of a subtropical grassland soil in Australia with biochar application after 8.2 and 10 years of a first and second biochar addition, respectively. The authors showed a negative priming of SOC by decreased native SOC mineralization (by 18%), a higher retention of rhizodeposits and microbial necromass in microaggregates and the mineral fraction, and an increased microbial C use efficiency in biochar-amended grassland^10^. In addition, beyond its potential for long-term carbon stabilization, biochar can improve soil structure and water retention, especially in degraded or compacted subsoils^10,11^.

Regenerative Agriculture combines these elements—reduced tillage, permanent ground cover, and system-oriented nutrient cycling. Although multiple definitions exist, RA can best be defined as “an approach to farming that uses soil conservation as the entry point to regenerate and contribute to multiple ecosystem services”^11^.

RA is discussed as a strategy to increase soil organic carbon climate change mitigation and adaptation^12–14^. So far, there is only limited knowledge on the SOC sequestration potential of RA in temperate regions. A recent meta-analysis by Jordon et al.^15^ examined three core regenerative agriculture (RA) practices—conservation tillage, cover cropping, and ley-arable rotations—and found that reduced tillage and ley-arable systems led to slight but statistically significant increases in SOC. However, these increases were only detectable after an average study duration of 15 years^15^.

One key barrier to evaluating SOC sequestration in RA, and agricultural experiments in general, is the slow rate of change in total SOC stocks against the large background carbon pool, since it typically requires monitoring over decadal timescales^16,17^. To address this limitation, researchers increasingly employ short-term biological indicators (e.g. microbial biomass carbon, CO_2_-C respiration, water-extractable carbon and nitrogen) that respond more quickly to management changes^18–20^. These indicators reflect microbial activity, labile carbon dynamics and nutrient cycling, and have been shown to be sensitive to land use changes and soil management in the early stages of system transformation^21,22^.

This study investigates the relationship between SOC stock changes and early biological indicators in a three-year organic field experiment in Hesse, Germany. The trial compares a conventional organic control with two regenerative treatments: one combining reduced tillage and diverse cover cropping (here refered to as the Regenerative Agriculture treatments, RA), and one extending this system with deep placement of biologically activated biochar (RABC) to break up a subsoil compaction layer and enable, in theory, root growth to greater depths. The study aims to assess whether early biological responses can serve as reliable indicators of SOC stock developments and to identify which soil biological parameters are most responsive during the initial transition phase under temperate organic conditions.

We hypothesized that:

1. Employing the combined regenerative agricultural (RA) practices “reduced tillage” and “cover cropping” will enhance early indicators.
2. The addition of biochar will enhance SOC accumulation and reinforce early (microbial) indicators for SOC stock increases as compared to RA alone.

## Material and methods

### Study site

**Fig. 1:**
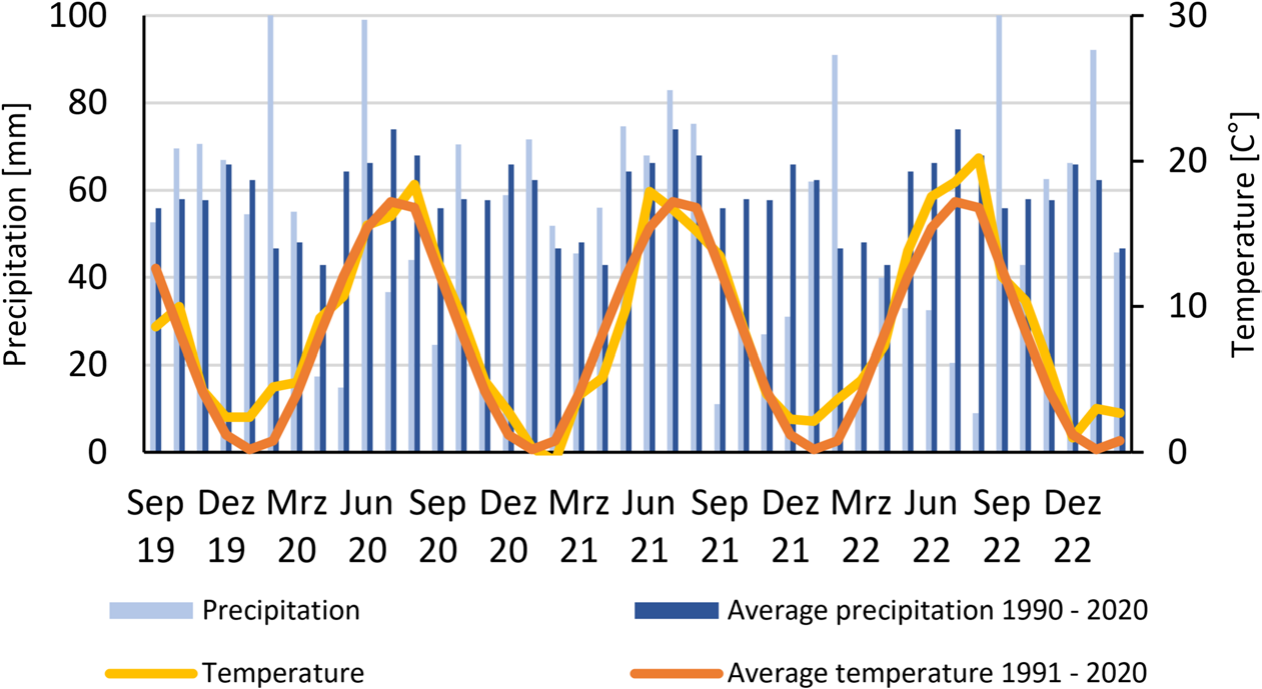
Monthly precipitation (bars, left axis) and temperature (lines, right axis) from September 2019 to February 2023. Light blue bars and yellow line represent observed precipitation and temperature during the study period; dark blue bars and red–orange line show long-term monthly means for 1990–2020 (precipitation) and 1991–2020 (temperature), respectively.

The on-farm research was conducted 2020–2023 on a mixed farm with dairy cows, arable crops and field vegetables that has been managed organically for 32 years. The farm is located in Gilserberg, Hesse, Germany, (50°57′10.8"N 9°03′40.9"E) at an elevation of 360 m above sea level. Prior to the experiment start in 2020, the crop rotation of the field included a two-year ley of clover-alfalfa-grass, followed by winter spelt, silage maize, triticale-pea-mixture, faba bean, winter rye, and summer oat. The average annual temperature during the time of the experiment was 9.2 °C with an average annual precipitation of 440 mm (+ 0.7 °C warmer and 12% drier than the regional 1991–2020 average; Fig. 1).

The soil type is classified as a Luvisol, with a sandy loam/loam (sL-L) texture containing 10% clay.

### Experimental setup and management

The concept for the field experiment was developed collaboratively by a group of farmers, advisors, and researchers. This group defined the regenerative agriculture practices and the crop rotation scheme for the trial, aiming to represent a practical yet comprehensive implementation of RA principles under organic farming conditions. While the conceptual orientation drew inspiration from the three-step RA framework proposed by Kurth et al.^23^, the experimental design was adapted to the specific context of organic agriculture in Central Europe.

Notably, several practices suggested by Kurth et al.^23^ are already standard in certified organic farming and were thus implemented across all treatments, including the control. These include routine soil analysis and nutrient balancing, moderate cover cropping, the use of biofertilizers (Table 2), and legume-based crop rotations. As such, they cannot be considered differentiating features between conventional organic and regenerative organic systems in this study.

To evaluate the added value of regenerative intensification, the RA treatments focused on practices that go beyond the organic baseline and are typically considered more advanced. These included deep subsoiling with minimal soil disturbance to alleviate compaction caused by a plough pan formed under previous conventional tillage, interseeding/intercropping (= nurse seeding;^24^) and the use of diverse and intensive cover cropping (Table 1). Treatment RABC extended RA by the one-time application of 4 t ha^-1^ biologically activated biochar with a subsoiler to a depth of approximately 30 cm (Table 2).

**Table 1:** Management summary of the fully randomized field trial specified for the three treatments Control (traditional Hessian organic farming system), RA (Regenerative Agriculture farming system) and RABC (Regenerative Agriculture with Biochar) over the three years 2020 to 2022.

Treatment	Control	RA & RABC
Tillage		
Annual crops	Annual full inversion 18–21 cm with a mouldboard plough	Annual shallow mixing to 5 cm (Rototiller)
Seedbed	Power Harrow, 5 cm	Rototiller, 5 cm
Subsoiling		Deep loosener 30 cm after first and second annual crop
Cover crop establishment	Stubble tillage 5–10 cm and seedbed preparation with Power Harrow	Rototiller, 5 cm
Green cover termination	Full inversion 18–21 cm (October, before Wheat; November/December before faba bean and silage maize)	Rototiller, 5 cm, 3–4 weeks before seeding; Rototiller, 5 cm while seeding
Equipment	Stubble tillage = 3 m Lemken Smaragd 9 (LEMKEN GmbH & Co. KG, D-46519 Alpen)	Rototiller = 3 m Geohobel seed drill combination (Rath Maschinen, A-9422 Maria Rojach)
	Mouldboard plough = 4-furrow Kuhn Master 113 (Kuhn S.A., F- 67,700 Saverne)	Deep loosener = 3 m Yeomans Plow with a 60 cm tine spacing (Yeomans Plow Co., Queensland Au-4075)
	Power Harrow = 3 m Lemken Saphir 7 seed drill combination (LEMKEN GmbH & Co. KG, D-46519 Alpen)	
Weed Control		
Spring tine weeder	Four passes in faba bean and silage maize, two passes in winter wheat	One pass in all annual crops
Rotary Hoe		One pass in all annual crops
Interrow cultivator	Two passes in silage maize	One pass in silage maize
Green Cover		
Cover crop	No winter-hardy mixture before silage maize: 60% white mustard (*Sinapis Alba*), 40% oilseed radish (*Raphanus sativus*)	Partially winter-hardy mixture before silage maize containing: Alexandrian clover (*Trifolium alexandrinum*), field pea (*Pisum sativum*), safflower (*Carthamus tinctorius*), crimson clover (*Trifolium incarnatum*), persian clover (*Trifolium resupinatum*), phacelia (*Phacelia tanacetifolia*), ramtill (*Guizotia abyssinica*), rye (*Secale cereale*), red clover (*Trifolium pratense*), alsike clover (*Trifolium hybridum*), serradella (*Ornithopus sativus*), common vetch (*Vicia sativa*), sunflower (*Helianthus annuus*), sorghum (*Sorghum bicolor*) tillage radish (*Raphanus sativus var. longipinnatus*), white clover (*Trifolium repens*), hairy vetch (*Vicia villosa*), flax (*Linum usitatissimum*), rye gras (*Lolium Perenne*)
Companion crop		Mixture sown together with faba bean (nurse crops): Oat (*Avena sativa*), buckwheat (*Fagopyrum esculentum*), flax (*Linum usitatissimum*), ryegrass (*Lolium perenne*), camelina (*Camelina sativa*), phacelia (*Phacelia tanacetifolia*), white mustard (*Sinapis alba*), coriander (*Coriandrum sativum*), tagetes (*Tagetes spp.*), calendula (*Calendula officinalis*), safflower (*Carthamus tinctorius*), Dill (*Anethum graveolens*)
Undersown crop		Mixture sown with a spring tine harrow at the 4-leaf stage of silage maize (nurse crops): Red fescue (*Festuca rubra*), white clover (*Trifolium repens*)

**Table 2:** Specifications for organic fertilizers applied in all three treatments and biochar inserted in the RABC (Regenerative Agriculture with Biochar) treatment only. Ntot = total nitrogen, Nmin = mineral nitrogen, P = phosphorus, K = potassium, Mg = magnesium, OM = organic matter, DM = dry mass, Corg = organic carbon and H/Corg = hydrogen organic carbon ratio.

Type	Value	Specification
Liquid cattle slurry	Application	Spring-application in Silage Maize and Winter Wheat conducted in accordance with the Hessian fertilization requirement determination guidelines, which consider humus content, previous crop, previous fertilizer and crop uptake. Annual average input except in years of faba bean (kg ha^-1^ a^-1^)
	N_tot_/N_min_	121.1/61.2
	P/K/Mg	22.3/171.6/21.2
	OM	1902.2
	Producer	Farm-own product
Composted manure	Application	Winter-application in all Treatments. Annual average input (kg ha^-1^ a^-1^)
	Ntot/Nmin	68.2/0.9
	P/K/Mg	39/78/19.5
	OM	3.350
	Producer	Farm-own product
Biochar (only for RABC)	Application	After first annual crop during subsoiling in RABC treatment. Injected at a depth of 30 cm with a line spacing of 60 cm. Total input (kg ha^-1^)
	Type	European Biochar certified (EBC-AgroBio) biological activated biochar
	Additives	Acid mixture (pH 3.5) of: Molasses, lactic acid bacteria, photosynthetic bacteria, and yeasts
	DM	2480 (62%)
	C_org_	2160
	N_tot_	0.01
	H/C_org_	0.34
	Producer	Carbuna AG, D-87700 Memmingen, Germany

The Control treatment followed conventional regional organic farming practices, including inversion ploughing and cover cropping with annual cover crops before maize cultivation.

**Fig. 2:**
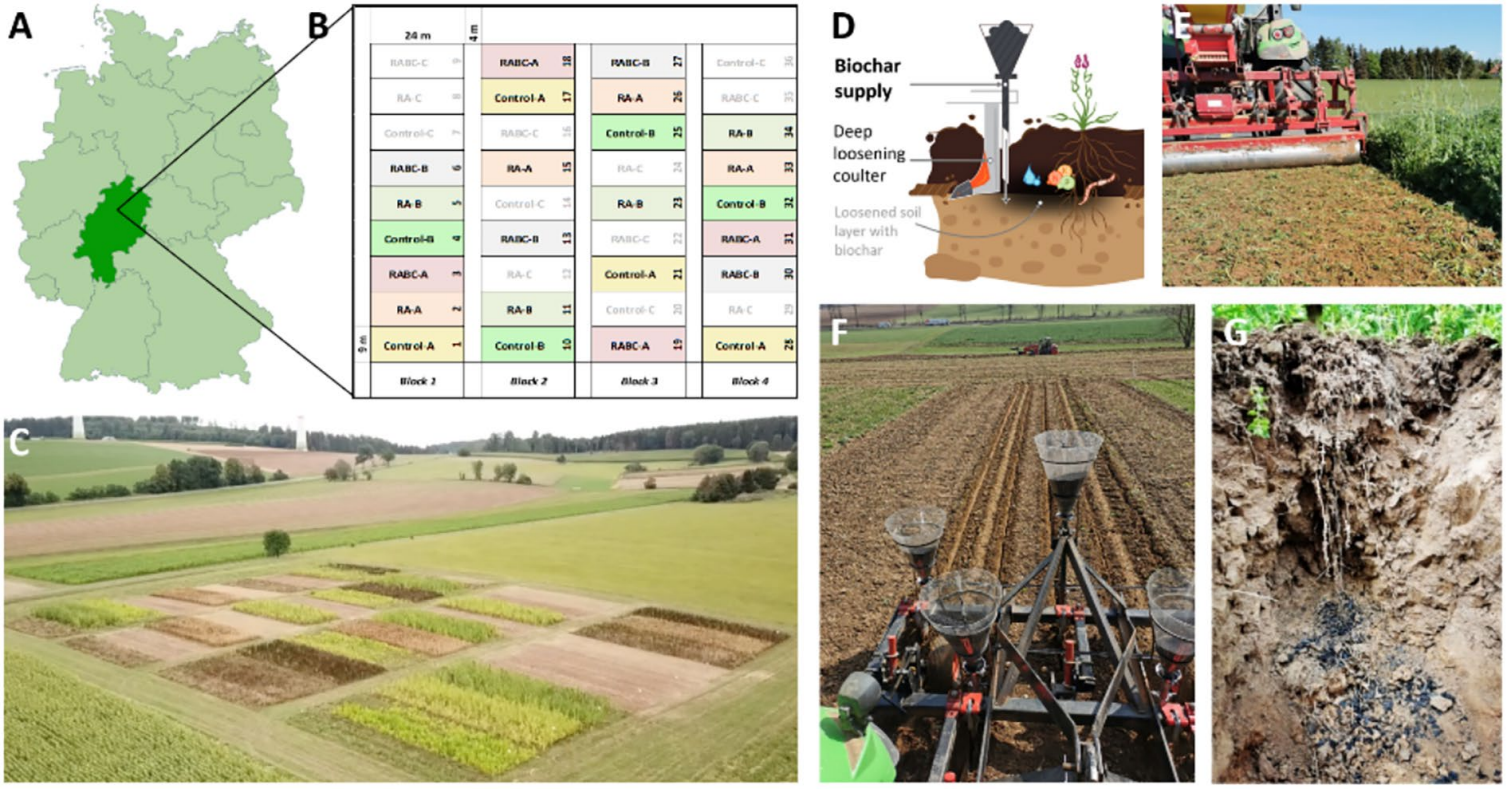
Illustration of the fully randomized field trial at Gilserberg. (**A**) Location in Hesse, Germany and (**B**) setup of the fully randomized field trial with the three treatments (Control = traditional Hessian organic farming system, RA = organic Regenerative Agriculture farming system, and RABC = organic Regenerative Agriculture with Biochar) and the three crop rotation cycles (**A**–**C**) in the four blocks (plot size 9 × 24 m). (**C**) Field trial in August 2020. (**D**) Schematic illustration of the biochar injection technique into 30 cm depth with a subsoiler. (**E**) Incorporation of cover crops in RA and RABC with a Geohobel. (**F**) Biochar injection in the RABC plot and (**G**) biochar in 30 cm depth under faba bean. Copyright: map (A): David Liuzzo, https://kurzlinks.de/6oac; foto (**C**): ARD-aktuell/tagesschau.de.

A fully randomized field experiment with 36 plots was conducted, comprising three treatments (Control, RA, RABC), three crop rotation cycles (A-C) and four replicates (Fig. 2). All treatments followed the same three-year crop rotation: faba bean (*Vicia faba*), winter wheat (*Triticum aestivum*), and silage maize (*Zea mays*). To account for rotation phase effects, the starting crop varied for the rotation cycles (A = faba bean, B = winter wheat and C = silage maize), resulting in a balanced design with each treatment represented at all rotation entry points.

For this study, only the plots with crop rotations A and B where used for analysis, due to poor development of silage maize in crop rotation C in 2020 as a result of rodent damage and an extremly dry year.

### Soil sampling and processing

Soil sampling was conducted for the first time in March 2020 and was repeated in March 2023, at least three months after the last soil disturbance, to minimize the influence of recent tillage on soil properties. For both the initial and final sampling events, three randomly selected soil cores were taken per plot, each georeferenced using real-time kinematic (RTK) positioning to ensure consistent spatial accuracy across years. The cores were spaced 10–30 cm apart, and extracted using a soil percussion core probe measuring 100 cm in length and 6 cm in inner diameter. At the second sampling time in the RABC treatment, cores were taken specifically between the biochar injection bands to avoid direct sampling of the amended zones. Samples were divided into five increments (0–10 cm; 10–30 cm; 30–50 cm; 50–70 cm; 70–100 cm) in accordance with the German Agricultural Soil Inventory^25^. Correction for core compaction and stretching was performed following a profile-specific approach, adapted from Walter et al.^26^. Compaction was allocated to the 30–100 cm section. In the case of soil core stretching, deviations ≤ 10 cm were distributed over the 0–30 cm depth, while deviations > 10 cm were allocated to the 0–50 cm segment, reflecting a modified application of the correction logic described by Walter et al.^26^. Soil samples were air-dried and sieved to 2 mm for carbon analysis. In 2023, half of the fresh sample material from the two upper increments was stored at 4 °C until further processing for MBC analysis.

### Soil sample analysis

MBC was determined after chloroform fumigation of 17.5 g fresh homogenized soil with ethanol-free chloroform in a desiccator for 24 h^27,28^. Extraction was performed using 0.01 M CaCl2, horizontal shaking for 30 min at room temperature and followed by filtration using MN 615 filter paper^29^). Extracts were stored at − 20 °C until analysis. Dissolved organic carbon (DOC) was measured using a LiquiTOC analyzer (Elementar Analysensysteme GmbH, Hanau, Germany). MBC was calculated as the difference in DOC between fumigated and non-fumigated samples, using an extraction coefficient (kₑC) of 0.45^30^.

The dried and sieved soil samples were used for the subsequent analysis of indicators for slow and fast carbon stock changes. The concentrations of the three carbon fractions total organic carbon, residual oxidisable carbon and total inorganic carbon (i.e., TOC400, ROC600, and TIC900, respectively) were analysed using smart combustion (DIN 13878) with a SoliTOC analyser (Elementar Analysensysteme GmbH, Hanau, Germany). Bulk density was determined for each soil core and depth increment based on the fine soil fraction, following the approach described by Poeplau et al.^31^. As the rock fragment content in all samples was below 2 vol. %, their Eq. 9 was applied^31^.

Soil organic carbon (SOC) stocks were calculated using the equivalent soil mass (ESM) approach to account for changes in bulk density over time. Specifically, Eqs. 5 to 7 from Fowler et al.^32^ were applied, which account for both changes in soil mass and SOC concentration across sampling years. The calculations were conducted using the publicly available tool developed by Haden et al.^33^, which incorporates spline interpolation and standardizes SOC stocks to a common soil mass baseline. The samples of the 0–10 cm and 10–30 cm increments were combined and further analysed for the following indicators. Water-extractable organic carbon (WEOC) and water-extractable organic nitrogen (WEON) were measured according to Guigue et al.^34^. Carbon Dioxide Respiration (CO_2_-C) within 24 h after rewetting was measured according to Haney et al.^35^ with an Infra Red Gas Analyser LI-COR LI-7000. The Haney Soil Health Score (SHS) was calculated using the Eq. 5 as developed by Haney et al.^22^, which integrates CO_2_-C, WEOC, and WEON.

### Statistical analyses

Statistical analyses were conducted using R (version 4.2.2;^36^) via the RStudio integrated development environment (RStudio, version 2023.06.0 + 421; available at https://posit.co/products/open-source/rstudio/).

To assess treatment effects on 2020–2023 changes in SOC concentrations and stocks, BD, CO_2_-C, WEOC, WEON, and SHS, as well as on MBC based on 2023 data only, stepwise model selection based on Akaike’s Information Criterion (AIC) was applied within a linear modeling framework^37^. For each response variable, a null model including only the intercept and a full model with fixed effects for *Treatment, Rotation, Replicate, Pseudoreplicate, Clay, Silt,* and *Sand* content was constructed. The *step()* function was used to perform forward and backward selection to identify the most parsimonious model^38^. Rotation (A and B) was included as a fixed effect and considered during model selection, but the two rotations were not analyzed separately in the statistical models.

Final models were evaluated using ANOVA, and estimated marginal means (EMMs) were calculated using the *emmeans R package* to enable pairwise comparisons among treatments^39^. For change of SOC concentration, SOC stocks, and BD, models were fitted separately for each depth increment (0–10 cm, 10–30 cm, 30–50 cm, 50–70 cm, 70–100 cm) and for the cumulative 0–100 cm profile. For MBC, models were fitted for the 0–10 cm and 10–30 cm layers and additionally aggregated for the 0–30 cm depth. In order to meet model assumptions of normality and homoscedasticity, MBC data were log-transformed prior to analysis. Residual diagnostics were conducted using graphical methods (residuals vs. fitted and Q-Q plots) and statistical tests (Shapiro–Wilk and Breusch-Pagan tests) to assess normality and homoscedasticity of residuals. Where necessary, model adjustments such as log-transformation of the response variable, heteroscedasticity-consistent standard errors, and robust linear regression were applied to address violations of normality and homoscedasticity assumptions. In the Results section, treatment effects are generally reported as means across Rotations A and B ± standard error (SE), along with *p*-values, unless stated otherwise. Data visualization was performed using *R package ggplot2*, including treatment-specific means and standard errors as well as depth-wise trend representations where applicable^40^. To illustrate potential rotation-specific trends, Rotations A and B were displayed separately in figures of SOC concentration, bulk density and SOC stocks although they were not treated separately in the statistical analysis. To explore potential relationships among soil carbon and biological indicators, Pearson correlation coefficients were calculated between changes in SOC stocks, MBC, CO_2_-C respiration, WEOC, WEON, and the Soil Health Score (SHS). The correlation analysis was conducted using the *cor.test()* function in R (method = “pearson”).

## Results

### SOC concentration, BD and SOC stocks

**Fig. 3:**
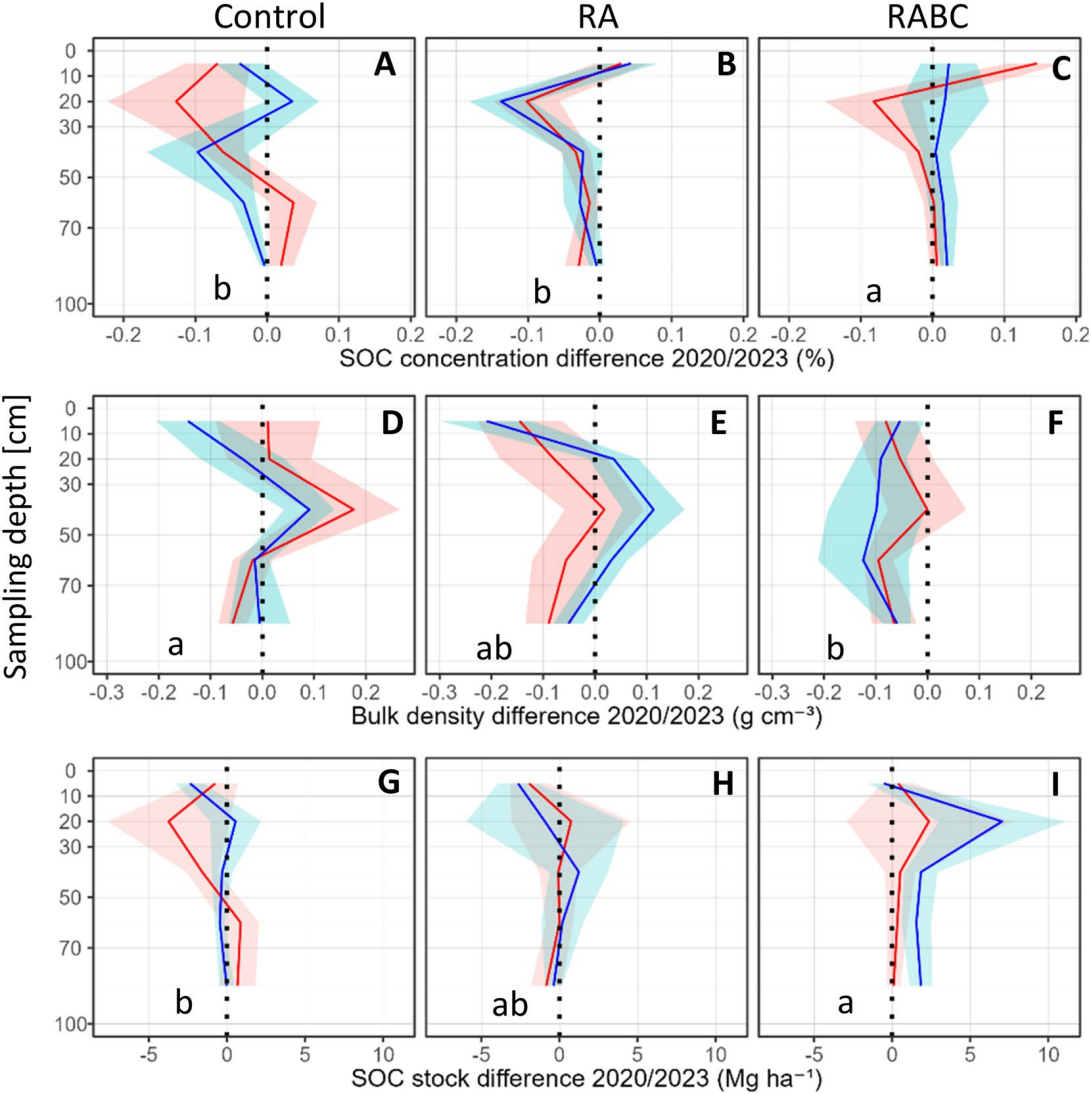
Profile depth differences in SOC concentrations (**A–C**), bulk density (**D–F**) and SOC stocks (**G–I**) for three contrasting agronomic-rotational treatments: standard organic agronomy (Control), a novel Regenerative Agriculture agronomic strategy (RA) and the novel RA plus injection of Biochar at 30 cm (RABC). The dotted lines indicate ‘normalised’ initial (time-zero) measured values in 2020, whilst the coloured solid lines (Rotation A (red: faba bean – winter wheat – silage maize) and Rotation B (blue: winter wheat –silage maize – faba bean) indicate with mean differences detected at the second coring in 2023. The treatments between time zero (2020) and resampling in 2023 (treatments x reps x cores x depth segments = 360). The halos around the mean lines show the standard error. Lowercase letters denote statistically significant differences between treatments across both rotations (*p* < 0.05).

Across the full 0–100 cm profile, SOC concentrations and SOC stocks remained largely unchanged in the Control and RA treatments, with non-significant declines in concentration from 1.00% ± 0.03 to 0.97% ± 0.03 (Control) and 1.01% ± 0.03 to 0.97% ± 0.03 (RA), and corresponding stock changes of -0.71 ± 0.65 Mg C ha^−1^ and -0.46 ± 0.65 Mg C ha^−1^ (both non-significant as compared to the starting year). In RABC, SOC concentration increased slightly over the same profile depth (1.01% ± 0.03 to 1.02% ± 0.03), with a significant difference to Control (p = 0.018) and RA (p = 0.038), and was accompanied by a significant gain in SOC stocks of + 2.24 ± 0.68 Mg C ha^−1^ (p = 0.035 vs Control). These changes occurred in parallel with a decrease in BD, which was non-significant in Control (− 0.0017 g cm^−3^, ≈ 0.1%) and RA (− 0.0417 g cm^−3^, ns, ≈ 2.7%), but significant in RABC (− 0.0709 g cm^−3^, p = 0.046, ≈ 4.5%; Figure 3 and Table 3).

**Table 3:** Pairwise treatment contrasts for changes in SOC concentration (%), bulk density (g cm^−^3), and SOC stocks (Mg C ha^−1^) between 2020 and 2023 across selected soil depths. Only results from models in which *Treatment* was retained as an explanatory variable following stepwise model selection are shown. Values represent estimated mean differences ± standard error (SE). Significance levels refer to treatment contrasts within the final models. Degrees of freedom (DF) and adjusted R2 values are reported for each model. Significance codes: *p* < 0.05 (*), *p* < 0.1 (.), not significant (ns).

Depth [cm]	Parameter	Contrast	Mean ± SE	Signif	Model	DF	R2_adj
0–100	SOC conc. difference (%)	Control—RA	− 0.005 ± 0.018	ns	~ *Treatment* + *Clay*	356	0.026
		Control—RABC	− 0.048 ± 0.018	*			
		RA—RABC	− 0.043 ± 0.018	*			
0–10	SOC conc. difference (%)	Control—RA	− 0.079 ± 0.040	ns	~ *Treatment* + *BD_Change*	68	0.146
		Control—RABC	− 0.138 ± 0.039	**			
		RA—RABC	− 0.058 ± 0.040	ns			
70–100	SOC conc. difference (%)	Control—RA	0.028 ± 0.013		~ *Treatment* + *Clay*	68	0.07
		Control—RABC	− 0.007 ± 0.013	ns			
		RA—RABC	− 0.035 ± 0.013	*			
0–100	BD difference (g cm^−3^ )	Control—RA	0.040 ± 0.029	ns	~ *Replicate* + *Silt* + *Treatment*	350	0.064
		Control—RABC	0.069 ± 0.029	*			
		RA—RABC	0.029 ± 0.029	ns			
30–50	BD difference (g cm^−3^ )	Control—RA	0.080 ± 0.073	ns	~ *Sand* + *Treatment*	68	0.116
		Control—RABC	0.202 ± 0.073	*			
		RA—RABC	0.123 ± 0.073	ns			
50–70	BD difference (g cm^−3^ )	Control—RA	− 0.005 ± 0.051	ns	~ *Treatment*	69	0.036
		Control—RABC	0.093 ± 0.051	ns			
		RA—RABC	0.098 ± 0.051	ns			
0–100	SOC stock difference (Mg C ha^-1^)	Control—RA	0.688 ± 0.650–70	ns	~ *Replicate* + *Treatment*	529	0.025
		Control—RABC	− 0.922 ± 0.675	ns			
		RA—RABC	− 1.610 ± 0.675	*			
0–10	SOC stock difference (Mg C ha^-1^)	Control—RA	0.598 ± 0.906	ns	~ *Replicate* + *Treatment* + *Pseudoreplicate* + *Rotation*	98	0.13
		Control—RABC	− 1.306 ± 0.906	ns			
		RA—RABC	− 1.904 ± 0.906				

In the 0–10 cm depth increment, SOC concentrations developed differently across treatments: Control showed a non-significant decline (1.45% C ± 0.05 to 1.40% C ± 0.05), RA a slight, non-significant increase (1.44% C ± 0.05 to 1.47% C ± 0.05), and RABC the strongest increase (1.44 C % ± 0.05 to 1.53% C ± 0.05), which differed significantly from Control (p = 0.0023). These changes in concentration were not reflected in significantly different SOC stock values at this depth. BD in the topsoil also decreased in all treatments, but treatment differences were not statistically significant.

Between 10 and 50 cm depth, SOC concentrations declined slightly across treatments (all ns). In the 30–50 cm layer, this was accompanied by a marked change in BD: changes over time were 0.1337 g cm^−3^ in Control, 0.0657 g cm^−3^ in RA, and − 0.0497 g cm^−3^ in RABC, corresponding to approximately + 8.5%, + 4.2%, and -3.2% of the profile mean, respectively. The difference between Control and RABC was significant (*p* = 0.034), while other contrasts were not (*p* ≥ 0.25). SOC stock changes in this depth range were not significant.

Below 50 cm, SOC concentrations remained largely stable across treatments. In the 70–100 cm layer, however, changes in SOC concentration differed between treatments: RA showed a decline (0.10% ± 0.003 to 0.08% ± 0.003), while RABC showed an increase (0.10% ± 0.003 to 0.11% ± 0.003); the difference between RA and RABC was significant (*p* = 0.027), while neither differed from the Control. BD differences in this depth range remained below 0.1 g cm^−3^ (≤ 6% of the profile mean) and were not significant (*p* ≥ 0.14). No significant differences in SOC stocks were detected at this depth.

### Soil respiration (CO_2_-C)

**Fig. 4:**
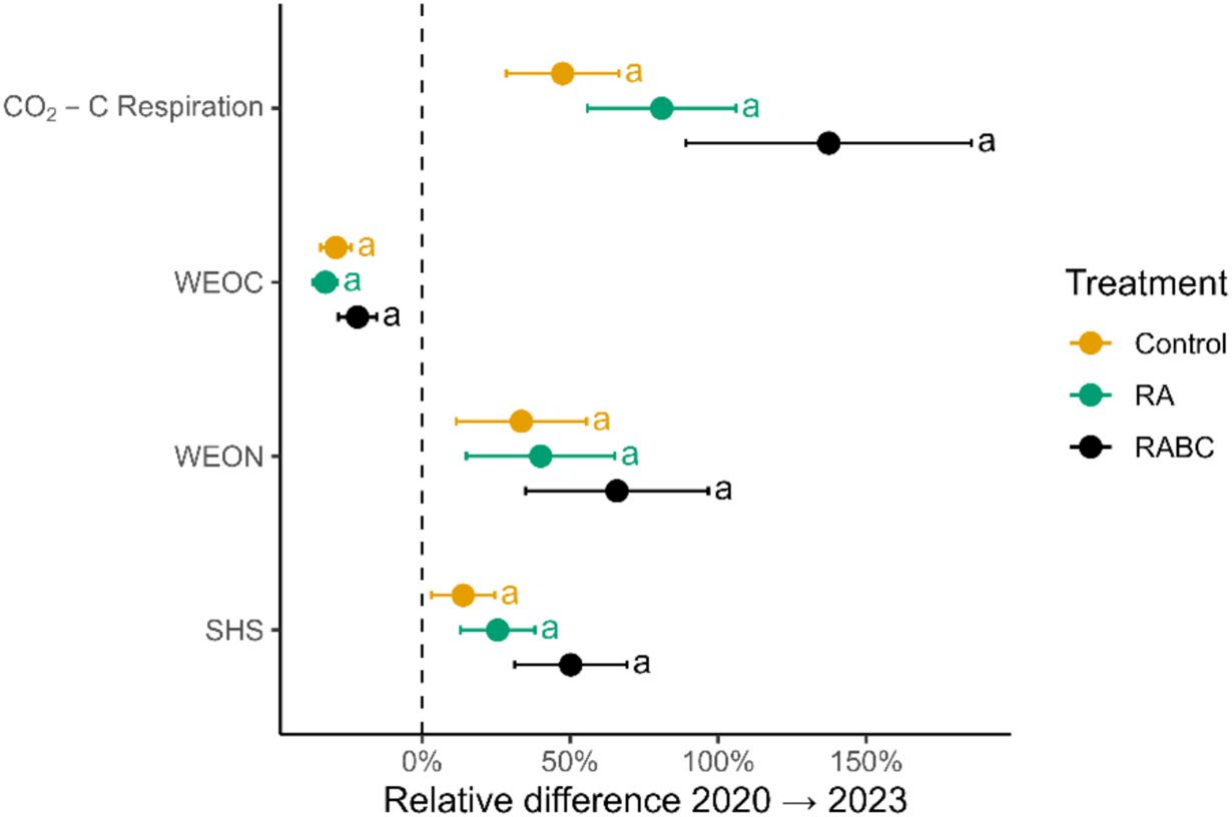
The dotted lines indicate ‘normalised’ initial (time-zero) measured values in 2020, whilst the coloured dots indicate the relative differences in % ± standard error detected at the second coring in 2023 for 0–30 cm soil depth (treatments x reps x rotations = 24) for the traditional Hessian organic farming system (Control), a novel Regenerative Agricultural system (RA) and the novel RA system plus Biochar. Lowercase letters denote statistically significant differences between treatments across both rotations (*p* < 0.05).

In all treatments, CO_2_-C respiration in the 0–30 cm soil layer increased relative to the measurements taken in 2020. The strongest absolute increase occurred in the RABC treatment, from 32.96 ± 3.58 mg C kg^−1^ in 2020 to 69.26 ± 8.73 mg C kg^−1^ in 2023 (+ 110%; Fig. 4). RA also increased substantially (+ 26.60 mg C kg^−1^), while the Control showed a moderate rise (+ 17.00 mg C kg^−1^). Although the factor treatment remained in the final model, ANOVA revealed no significant overall treatment effect (p = 0.129).

### Water-extractable organic carbon (WEOC)

WEOC declined from 2020 to 2023 in all treatments in the 0–30 cm soil layer. The RABC treatment had the highest WEOC in 2020, with 345.3 ± 13.33 mg C kg^−1^, but decreased to 269.0 ± 13.34 mg C kg^−1^ in 2023 (− 76.3 mg C kg^−1^; Fig. 4). RA showed the strongest decline from 330.9 ± 13.33 mg C kg^−1^ to 217.3 ± 6.85 mg C kg^−1^ (− 113.6 mg C kg^−1^). The Control dropped from 314.1 ± 13.33 mg C kg^−1^ to 217.3 ± 13.33 mg C kg^−1^ (− 96.8 mg C kg^−1^).

In the stepwise regression analysis, none of the potential explanatory variables (*Treatment, Rotation, Replicate, Clay, Silt, Sand*) significantly explained the variance in the WEOC change. The null model was retained (AIC = 125.94), and the ANOVA confirmed no significant treatment effect (*p* > 0.1).

### Water-extractable organic nitrogen (WEON)

WEON concentrations increased from 2020 to 2023 in all treatments within the 0–30 cm soil depth. RABC increased the most from 7.38 ± 0.71 mg N kg^−1^ to 10.43 ± 0.71 mg N kg^−1^ (+ 41%; Fig. 4). RA and Control increased + 2.24 mg N kg^−1^ (from 5.78 ± 0.71 mg N kg^−1^ to 8.02 ± 0.71 mg N kg^−1^) and by 1.27 mg N kg^−1^ (from 6.71 ± 0.71 mg N kg^−1^ to 7.98 ± 0.71 mg N kg^−1^), respectively. Clay content was the only explanatory variable retained in the final model, explaining a small but non-significant portion of variance (adj. R2 = 0.073, *p* = 0.114). Treatment did not significantly affect WEON change (ANOVA *p* = 0.20). No significant effects of treatment or rotation were found for WEON change (all *p* > 0.68).

### Soil health score (SHS)

In the 0–30 cm soil layer, the SHS of the treatments were not significantly different (*p* = 0.148; Fig. 4), but an increasing trend was observed in the RABC treatment (+ 50.2 ± 14.1) from 2020 to 2023. The Control treatment had an average SHS change of + 13.8 ± 14.1, while the RA treatment exhibited a higher SHS increase (+ 28.9 ± 15.2); however the difference was not statistically significant (*p* = 0.128).

### Microbial biomass carbon in 2023 (MBC)

**Fig. 5:**
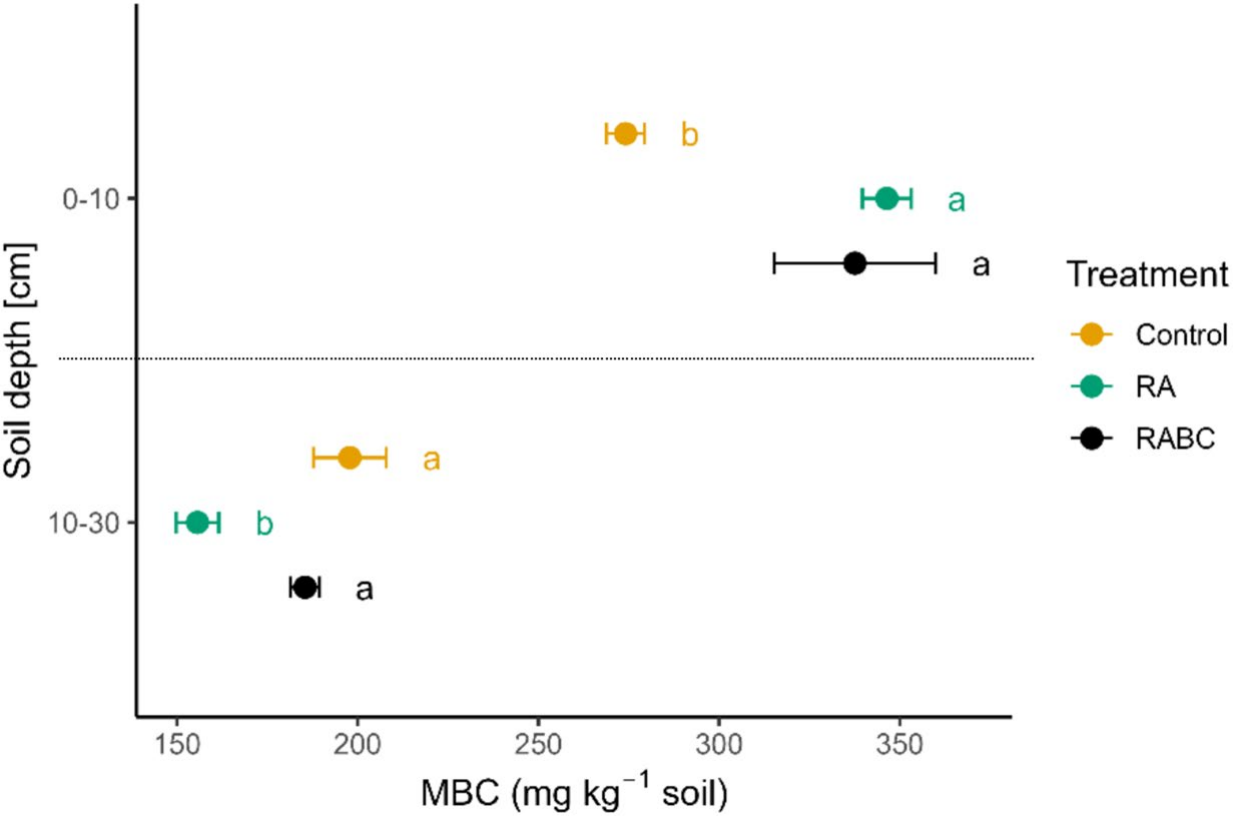
The coloured dots indicate the mean Microbial Biomass Carbon (MBC) ± standard error detected at the second coring in 2023 (0–10 cm and 10–30 cm; treatments × replicates × rotations = 24) for the traditional Hessian organic farming system (Control), a novel Regenerative Agricultural system (RA) and the novel RA system plus Biochar. Lowercase letters denote statistically significant differences between treatments across both rotations (*p* < 0.05).

A significant treatment effect on MBC over the mean of the two roations was observed for both soil layers, 0–10 (*p* < 0.001) and 10–30 cm (*p* < 0.001; Fig. 5). In the first layer, the Control treatment had the lowest MBC values, averaging 274 ± 13.8 mg C kg^−1^. The RA and RABC treatments showed significantly higher MBC levels of 346 ± 13.8 mg C kg^−1^ (p = 0.0012 compared to Control) and 338 ± 13.8 mg C kg^−1^ (*p* = 0.0051 compared to Control), respectively. RA and RABC were not different (*p* = 0.89).

The reverse was observed for the 10–30 cm soil layer, where the Control treatment exhibited the highest MBC levels with 205 ± 6.35 mg C kg^−1^ followed by the RABC treatment with 183 ± 6.14 mg C kg^−1^ and the RA treatment with 152 ± 6.18 mg C kg^−1^ (*p* < 0.001 compared to Control). The intermediate MBC value of the RABC treatment had a tendency to be lower than the Control (*p* = 0.081), but was significantly higher than that of the RA treatment (*p* = 0.0001). In addition to treatment, soil texture (clay and silt content), *Replicate*, and *Rotation* had significant effects on MBC.

Cumulating the topsoil layers to 0–30 cm soil depth, MBC showed a weak treatment effect (*p* = 0.087). The Control treatment had a mean MBC of 232 ± 11.3 mg C kg^−1^, while RA showed a slight but non-significant increase to 259 ± 11.3 mg C kg^−1^ (*p* = 0.22). The RABC treatment exhibited the highest MBC with 265 ± 11.3 mg C kg^−1^ and a marginal significant increase compared to the Control (*p* = 0.092). No significant difference was detected between RA and RABC (*p* = 0.91).

### Correlation analysis

Pearson correlation analysis revealed no statistically significant relationships among changes in SOC stocks, microbial biomass carbon, CO_2_-C respiration, water extractable organic carbon and nitrogen, or the Soil Health Score.

## Discussion

### Indicators of SOC development in the novel regenerative agriculture treatment

The RA treatment did not result in significant SOC stock increases over the three-year period. However, over longer time scales, such increases were detected with comparable management methods. For example Poeplau & Don^3^ reported over 50-year timescale and Jordon et al.^15^ over 30-year timescale that SOC increases of 0.3 Mg C ha^−1^ yr^−1^ can be achieved with cover cropping. Also, Krauss et al.^6^ reported SOC increases ranging from 0.09 to 0.27 Mg C ha^−1^ yr^−1^ under reduced tillage in organic farming, compared to conventional ploughing, across a period of 8 to 21 years.

In line with our short-term results, Anuo et al.^41^ also found no increase in SOC stocks after five years of cover cropping, but observed significant changes in labile C fractions such as WEOC and free particulated organic matter (f-POM)—pointing to their potential as early indicators of shifts in SOC dynamics. This supports the hypothesis that root-derived C inputs and microbial processing, as proposed by Poeplau & Don^3^ and Cotrufo et al.^8^, play a key role in the initiation of SOC stabilization. Similiar early microbial responses were observed in our study, which also align with findings from Kim et al.^42^ and Muhammed et al.^43^, who reported increased MBC following cover cropping. Oberholzer et al.^44^ likewise found elevated MBC under reduced (shallow) tillage, attributing this to greater availability of labile C—a condition comparable to that in the 0–10 cm layer of both the RA and RABC treatments. The comparatively modest increase in SOC concentrations, alongside a more pronounced rise in MBC, resulted in a lower C-to-MBC ratio, indicating that microbial pools respond more rapidly to management change than bulk SOC stocks and that the system is still in transition^45^.

A possible explanation for this unexpected results may lie in the dynamics of microbial priming effects associated with cover crop inputs.

Firstly, it is plausible that cover cropping in our regenerative treatments triggered positive priming effects or N-mining, which enhanced the microbial decomposition of existing soil organic matter (SOM), thereby offsetting any potential gains from the cover crop-derived carbon input. So called ‘Positive priming’ effects are driven by an increase in microbial activity or microbial biomass in response to plant derived substrate addition and the associated tillage increasing oxygen can lead to intensified SOM decomposition^46^. In contrast, ‘Negative priming’ suppresses microbial breakdown of new plant derived substrates and/or an increase in microbial C use efficiency reducing SOM breakdown^46^.

Secondly, the quantity and chemical composition of plant biomass inputs from the different crops in the rotation may have been insufficient to provide the substrate required to promote soil organic matter formation. Priming effects depend on microbial biomass dynamics and plant–microbe interactions and are often triggered by small organic inputs^46–48^. These effects are typically induced by small additions of organic material^46^. Liang et al.^49^ demonstrated that long-term cover cropping does not necessarily lead to SOC increases unless a critical biomass input is reached. The required input appears to lie between 0.2 and 0.3 mg C g^−1^ soil, which corresponds to an aboveground biomass of approximately 0.7 and 1.1 Mg dry matter ha^−1^.

In late summer and autumn 2020 and 2021 at Gilserberg lower than average rainfall reduced cover crop establishment following the main crop from an expected 2 Mg dry matter ha^-1^ to less than 1 Mg dry matter ha^-1^. Even more severe and prolonged water deficits in 2022 (Fig. 1) reduced both main crop and cover crop yields to well below 0.7 Mg dry matter ha^−1^ threshold of residue entering the soil, resulting in SOC reductions in all treatments.

To enable SOC formation to sequester carbon from the atmosphere and improve soil properties, cover cropping systems must be designed to supply more carbon of different forms into the soil than is lost through respiration. This includes choosing appropriate species, optimizing sowing time, and ensuring adequate growth conditions. In our system, only maize provided a sufficiently long window for robust cover crop establishment, whereas faba bean and winter wheat left limited time for growth. This likely contributed to the partly divergent development of SOC concentrations observed between rotation A and B (Fig. 3), where differences in cover crop, main crop and nurse crop biomass due to crop rotation and drought sensitivity may have influenced carbon input levels.

Thirdly, residue quality—particularly the C:N ratio—may have influenced the magnitude and direction of priming responses. Plant residue C:N ratios shape microbial nutrient supply and community functioning, and increasing senescence can promote N limitation and immobilization^50,51^. In our system, such quality effects could have contributed to short-term variability in microbial responses without translating into measurable SOC stock gains.

Together, these results indicate that short-term SOC stock assessments may miss early microbial and biochemical shifts under regenerative management.

The high variability observed in the SOC data further supports the interpretation of an ongoing dynamic process. According to the microbial efficiency-matrix stabilization (MEMS) framework^8,52^, labile carbon inputs from roots and cover crops can enhance stable SOC formation through microbial mediated pathways. The underlying process for this SOC formation involves rapid microbial growth and turnover, during which plant-derived carbon is transformed into microbial residues (necromass), which have been shown to contribute approximately 50–80% of persistent, mineral-associated SOC^53^. Therefore, increases in MBC could be viewed as sensitive early indicators for upcoming SOC stabilization under regenerative management. This interpretation is further supported by the absence of measurable SOC stock accumulation in the topsoil of the RA treatment, despite increased MBC and positive trends in SOC concentration. It emphasizes the importance of accounting for structural soil changes, which may mask short-term SOC dynamics if not corrected for the soil mass using ESM. In contrast to findings by Krauss et al.^6^, who reported that organic management, cover cropping, and reduced tillage typically enhance SOC stock accumulation only in the topsoil (down to 10–15 cm), our study observed increases only in SOC concentrations, not SOC stocks, at this depth. After applying the ESM method, apparent SOC increases in the topsoil were largely attributable to reduced bulk density rather than net carbon inputs. When SOC stocks were expressed on an equivalent soil mass basis, the remaining stock differences were concentrated in the 10–30 cm layer, reflecting a shift in the depth distribution of comparable soil masses rather than a direct downward translocation of carbon. This finding underscores the need to segment soil cores into fine depth intervals and determine bulk density for each segment individually, especially when assessing SOC changes under changing management regimes, as proposed by Fowler et al.^32^. Moreover, whole-profile assessments are affected by high natural variability and reduced statistical power with increasing depth^54^. It also calls the validity of many SOC stock assessments where such corrections are not applied, potentially leading to misinterpretation of depth-related C dynamics^32,33^.

Taken together, the findings of this study show that, despite positive microbial and structural signals, the RA treatment did not translate into statistically detectable gains in SOC stocks within the three-year period. This is not surprising, and concurs with simulations with soil carbon models such as the Rothamsted Carbon model (RothC)^55^ that show that SOC stock change detection requires about a decade.

These time scales in a continously changing climate are making it difficult to make best practice recommendations. Wiltshire & Beckage^56^ used the RothC model to demonstrate that, for Vermont, U.S., integrating climate change into projections of SOC sequestration leads to a net SOC loss by 2099—even under the best regenerative arable farming practices—due to increased SOC mineralisation due to rising soil temperatures. They showed that only the most intense regenerative practices including perennials such as rotational grazing and old-growth afforestation could raise long-term SOC stocks.

### Effect of biochar on SOC accumulation and early indicators

This study found that increases in SOC stocks occurred only when biochar application was included as part of the regenerative management system. Under the RABC treatment, SOC stocks increased on average by + 0.75 Mg C ha^−1^ yr^−1^. Blanco-Canqui et al.^9^ reported a mean annual SOC increase of approximately + 2.35 Mg C ha^−1^ yr^−1^ over six years after applying 7.25 Mg C ha^−1^ biochar, in a system combining no-till and cover cropping. Although the biochar application rate in our study was lower (2.2 Mg Biochar-C ha^−1^) compared to other studies^57,58^, the concentrated placement at 30 cm depth, to open the compacted plough layer in this depth for root growth, likely enhanced its effectiveness—an effect that has also been observed in other studies involving deeper biochar application^59–61^. This is particularly evident when comparing MBC in the 10–30 cm layer between RA and RABC. In RA, MBC was significantly lower than in the control, supporting concerns raised by Krauss et al.^6^ about potential SOC losses under reduced tillage due to limited incorporation of organic residues. In RABC, however, the reduction in MBC was mitigated. Although the sampled depth did not cover the injection bands directly but rather the space between them, we still observed reductions in bulk density and increases in SOC in what had previously been a plough pan formed by conventional tillage.

A direct physical or dilution effect of the biochar can therefore be excluded. Unlike in Obia et al.^62^, where biochar was mixed directly into compacted zones and functioned as a structural soil amendment, the effects observed here are more likely due to indirect mechanisms. These likely include improved oxygen diffusion, water infiltration and, potentially as a consequence, enhanced root growth or stimulated biological activity in adjacent zones influenced by biochar presence^63,64^. Such mechanisms are supported by studies showing that biochar application can improve root penetration and root growth, soil aggregation and porosity, thereby enhancing microbial activity and nutrient cycling^9,60^. In addition, isotopic studies indicate that biochar can reduce native SOC mineralization (negative priming) and increase retention of new carbon inputs in aggregates and mineral-associated fractions^10,65,66^.

Deeper biochar placement has been shown to increase microbial biomass and reduce specific respiration (qCO_2_), indicating greater microbial efficiency and a shift toward SOC stabilization^67^. In our study, this response was only partly reproduced: although qCO_2_ increased under RABC, SOC stocks still increased, suggesting that SOC gains were driven primarily by increased root-derived carbon inputs and their physical stabilization in aggregates and mineral-associated fractions rather than by enhanced microbial carbon use efficiency, similar to observations by Ding et al.^68^. Comparable increases in microbial activity and MBC after biochar addition have been widely reported and are often associated with arbuscular mycorrhizal fungi^69–73^.

In the present study, pre-acidification of the biochar likely enhanced surface oxidation and nutrient retention^74,75^, potentially reinforcing these biological responses. The increase in the SHS in the RABC treatment (where samples were not taken directly in biochar application places but in-between), reflecting both elevated CO_2_-C respiration and a more favorable WEOC:WEON ratio, further supports enhanced soil biological functioning, accompanied by improved root penetration, in response to the combined effects of reduced tillage, cover cropping and strategically placed biochar. As SHS has been linked to microbial activity, organic matter accumulation and water retention^76^, the observed increase indicates that the RABC system is transitioning toward a more regenerative state.

### Requirements and constraints for SOC sequestration in regenerative agriculture

The results support the interpretation that the effectiveness of regenerative agriculture depends on the integration of multiple, complementary practices across system levels. In our study, the RA treatment implemented a broad spectrum of practices in line with the three dimensions of RA defined by Kurth et al.^23^. Focusing solely on cultivation practices, as is sometimes done in the literature, fails to capture the complexity of system-level interactions. Jordon et al.^15^ emphasized this point, showing that SOC responses vary strongly depending on combinations of practices and site-specific conditions. It is also important to recognize that soils with already high SOC stocks may approach a carbon saturation threshold, limiting their capacity to stabilize additional organic inputs^77^, although biochar amendment to a subtropical grassland soil has been shown to lift this ”SOC ceiling”^10^. In such cases and without biochar addition, even well-designed interventions like cover cropping or reduced tillage may yield only marginal SOC stock gains^78^—unless complemented by amendments that alter the stabilization environment, such as biochar or deep-rooted perennials^10,55,79^.

However, a major challenge remains in the practical implementation and economic viability of biochar use. The costs of the biochar in this study were approximately 800 € Mg^-1^ which is economically unfeasible under current market conditions. No significant yield improvements were observed during the three-year study period, and other studies have reported comparable findings in temperate climate zones and soils^15^, indicating that such high costs cannot be compensated through productivity gains alone. Here, the existing measuring, reporting and verification schemes such as the world biochar certificate (WBC, 2023 at Carbon Standards Interational, see https://www.carbon-standards.com/en/standards/) and the increasing global demand for certified, controlled biochar-CDR (carbon dioxide removal, see leaderboards at https://www.cdr.fyi/) may over time lead to cost reductions for farmers.

## Conclusions

RA improved bulk density and microbial biomass in topsoil but did not result in a significant SOC gain within the short period of three years. In contrast, combining RA with subsoil biochar application (RABC) led to a significant SOC stock increase (+ 2.24 Mg C ha^−1^), along with further improvements in soil structure and microbial biomass. As highlighted by Wiltshire^54^, RA may serve more to preserve existing SOC under climate stress, whereas only intensified strategies, like those defined by Kurth et al.^23^ as advanced regenerative implementations (biochar, rotational grazing, and agroforestry), offer true potential for net SOC accumulation. However, given the short observation period of only three years, no robust conclusions can yet be drawn regarding the long-term sequestration potential of regenerative practices.

### Outlook

To strengthen the role of regenerative agriculture in climate mitigation, broader system-based evaluations are needed. These should include long-term carbon balances, economic analyses, and integration into carbon certification schemes. A full carbon balance is necessary to assess the net CO_2_ mitigation effect of regenerative practices. Practical tools for monitoring SOC and soil health in a cost-efficient manner are also essential^80^. To ensure implementation at scale, both ecological and economic aspects, as well as the climate resilience of farming systems, must be considered.

In the context of this study, further investigation is planned within the project "PK-Boden-ABC," including a second SOC assessment in 2025 and detailed analysis of root systems using AI-assisted image profiling to assess below-ground carbon dynamics and stabilization mechanisms.

## Data Availability

The datasets generated and/or analysed during the current study are available from the corresponding author on request.
